# The Biocompatibility of a New Type of 45S5 Bioactive Graft in a Sheep Model: A Pilot Study

**DOI:** 10.7759/cureus.41521

**Published:** 2023-07-07

**Authors:** Erhan Okay, Ali Can Ozarslan, Özgür Başal, Hüseyin Cakıroglu, Sevil Yucel, Korhan Özkan, Mahmut Nedim Doral

**Affiliations:** 1 Department of Orthopaedics, Goztepe Research and Training Hospital, Istanbul, TUR; 2 Faculty of Chemical and Metallurgical Engineering, Department of Bioengineering, Yildiz Technical University, Istanbul, TUR; 3 Department of Orthopedics and Traumatology, Emsey Hospital, Istanbul, TUR; 4 Experimental Medicine Research and Application Center, Faculty of Medicine, Sakarya University, Sakarya, TUR; 5 Department of Orthopaedics and Traumatology, Medeniyet University Goztepe Training and Research Hospital, Istanbul, TUR; 6 Faculty of Medicine, Department of Orthopaedics, Hacettepe University, Ankara, TUR; 7 Department of Orthopaedics, Magnet Hospital, Ankara, TUR

**Keywords:** experimental sheep surgery, preclinical research, experimental animal model, biocompatibility, bioactive material

## Abstract

Background

Bone is a dramatically regenerating tissue with the ability to heal after trauma, although intensive surgical management is required to treat considerable damage. In this study, 45S5 bioactive grafts were prepared through the melt-quenched method in compliance with the guidelines on medical product requirements (MDD regulations; 93/42/EEC Annex-II section 3&4 and ISO standardizations; ISO 13485:2016) for bone repair and regeneration.

Methodology

After preparing the graft/scaffold, it was evaluated for biocompatibility according to the principles of “lSO 10993-6 2015 Biological evaluation of medical devices: Tests for local effects after implantation, Annex D ‘Test method for implantation in bone,’” “lSO 10993-2:2005 Biological evaluation of medical devices: Animal welfare requirements,” and “lSO 10993-12 2012 Biological evaluation of medical devices sample preparation rules and standards.”

Defects were created on the tibia of the right hind leg. The defects were filled with 3-mm bioactive granules, and a cylindrical polypropylene biocompatible material was used as a negative control. After 120 days, the sheep were sacrificed, and the tibia were analyzed.

Results

The results demonstrated the safety of 45S5 bioactive grafts. Histological evaluation showed no signs of pathological changes around the implant area. Hematoxylin and eosin sections demonstrated the presence of a few multinucleated giant cells, macrophages, and non-irritant mild fibrotic changes on the surface of the biomaterial.

Conclusions

45S5 bioactive glass was found to be biocompatible in a sheep model, demonstrating its capacity to promote bone consolidation while also justifying its further preclinical application as a bone-bonded material owing to the layer formation of the growing bone mineral.

## Introduction

Bioinert materials, which behave in a biologically inert manner in the living body, were first used by scientists and surgeons as alternatives to human tissue. The first generation of biomaterials was made of metals and polymers. However, these materials are usually biologically incompatible with living tissue after implantation [1]. A unique type of ceramic material called bioactive glass was invented as a second-generation tissue replacement in 1969 [2]. The bioactivity of bioactive glass has allowed tissue treatment approaches to be greatly expanded. In vivo studies have shown the absence of interfacial scar tissue between bioactive glasses and host bone, and bioactive glasses cannot be removed from the implant sites [3]. These observations resulted in the creation of different biomaterials known as bioactive materials that are used in implants and in the repair or replacement of bones, joints, and teeth [4]. Rapid regeneration of trabecular bone is achieved through 45S5 Bioglass with high bioactivity rates. The amount, structure, and biomechanical properties of the regenerated bone are identical to those of the original bone at the site after treatment with bioactive glass. Osteoconduction and osteostimulation are responsible for the quick regeneration of bone [5-8]. When bioactive glass interacts with body fluid, the formation of a biological hydroxyapatite (HA)-like mineral phase that mimics the inorganic phase in living bone occurs on the surface of bioactive glasses [9,10]. Bioactive glasses bond to the bone via the interface as a layer of growing bone mineral that has interdigitated with collagen fibrils generated by osteoblasts growing at the interface [11-13]. Bioactive glasses became an important graft material used worldwide for tissue regeneration and treatment in clinical applications due to their excellent features. Further, recent studies have found that bioactive glasses with particularly high levels of bioactivity can be used to activate genes to encourage the body to heal itself [14]. The use of bioactive glass is also common in orthopedic surgery to fill the defects that result after tumor surgery, bone fracture, or debridement due to osteomyelitis. This pilot study aims to evaluate the biocompatibility of 45S5 bioactive graft, a new type of bioactive graft, in a sheep model.

## Materials and methods

Graft preparation

Sterile-packaged, melt-quenched bioactive glass granules were produced according to the guidelines of the ISO 13485 quality management system. Briefly, the precursors required for the bioactive glass oxide component were melted at high temperatures and obtained in granular form by cold casting. Bioactive glass particles in the form of granules (particle size: 1-3 mm) were sieved using a suitable sieve. Subsequently, the granules were packaged and sterilized using ethylene oxide. Sterile-packed bioactive glass granules were stored at 5-35°C.

Animal study

Ethical approval was obtained from the local animal ethical committee of the Kırıkkale University of Scientific and Technological Research Center (approval number: 2018/10/58). Three healthy 12-month-old female Akkaraman ewes (weighing 25-27 kg) were obtained from the laboratory production unit of the university. The animals were kept in 12-hour light/dark-cycle cages programmed with standard humidity (30-70%) and temperature (20 ± 3°C). The animals were allowed free access to a standard diet and tap water until the study protocols began. All regulations were met throughout the experiments, and all experiments were performed in accordance with the Animal Welfare Act and the Guide for the Care and Use of Laboratory Animals.

Bone implantation tests were performed according to the principles of “ISO 10993-6 2016 Biological evaluation of medical devices: Tests for local effects after implantation, Annex D ‘Test method for implantation in bone.’” All surgical operations were performed under deep general anesthesia with monitoring of vital health parameters. For this purpose, 200 µg/kg xylazine HCl (2%, Alfazyne, Alfasan) and 10 mg/kg ketamine hydrochloride (Ketamine, Parke Davis, Eczacibasi) were intramuscularly administered. The implantation area, which was previously shaved and cleaned with an iodinated disinfectant, was closed with a sterile cervix along the median line at the level of the proximal tibia. After taking precautions in accordance with the rules of asepsis and antisepsis in the operating room environment, a 10-cm long skin incision was made on the right medial hind leg of the sheep, which was fixed in the ventrodorsal position on the heated operating table. The muscles of the region were dissected using the blunt dissection method with scissors, and the proximal end and metaphyseal region of the tibia were reached. Here, the periosteum was separated with the blunt end of the scalpel, and the resulting bleeding was buffered. Then, starting from the proximal end of the tibia, five holes with a diameter of 3 mm were created distally following each other at the same level. While drilling the hole, the drill speed was kept at the lowest level and washed with sterile distilled water when necessary. Afterward, a 45S5 bioactive graft material with a particle size of 2-3 mm was placed in the opened bone holes. After skin and muscle incisions were made in the contralateral tibia region as described above, five holes with a diameter of 3 mm were drilled into the bone, and for negative control purposes, 2.5 × 5-mm cylindrical polypropylene biocompatible material was placed in the holes. Subsequently, muscle and fascia tissues were sutured with catgut thread and the skin area was closed with 2/0 silk thread (Dogsan Sanayi, Istanbul, Turkey), wiped with iodinated disinfectant, and dressed using elastic surgical tape. After surgery, intramuscular 2.5 mg/kg enrofloxacin (Baytril 10%, Bayer Inc.) was administered for five days, and the skin incision wound bandage was daily renewed for three days. Any analgesic or pain reliever was not given to the animals because of their potential anti-inflammatory and regenerative effects on the experimentally induced bone defects. Following the surgical operation, intramuscular 2.5 mg/kg enrofloxacin (Baytril 10%, Bayer Inc.) was used for five days.

Histological and radiological evaluation

At 30 and 120 days after implantation, radiographic analysis was performed to evaluate bone filling and mineralization. Euthanasia was performed using intramuscular pentobarbital. The samples were fixed in 10% formalin for 24 hours. They were decalcified within a decalcification solution (Osteodec Bio-Optica). Each of the decalcified materials was cut into anteroventral directions. Tissues were dehydrated, infiltrated, and embedded in paraffin. Then, 5 µm thick specimens were prepared after cutting in a microtome and stained with hematoxylin and eosin (Sigma-Aldrich, Darmstadt/Germany).

## Results

**Figure 1:**
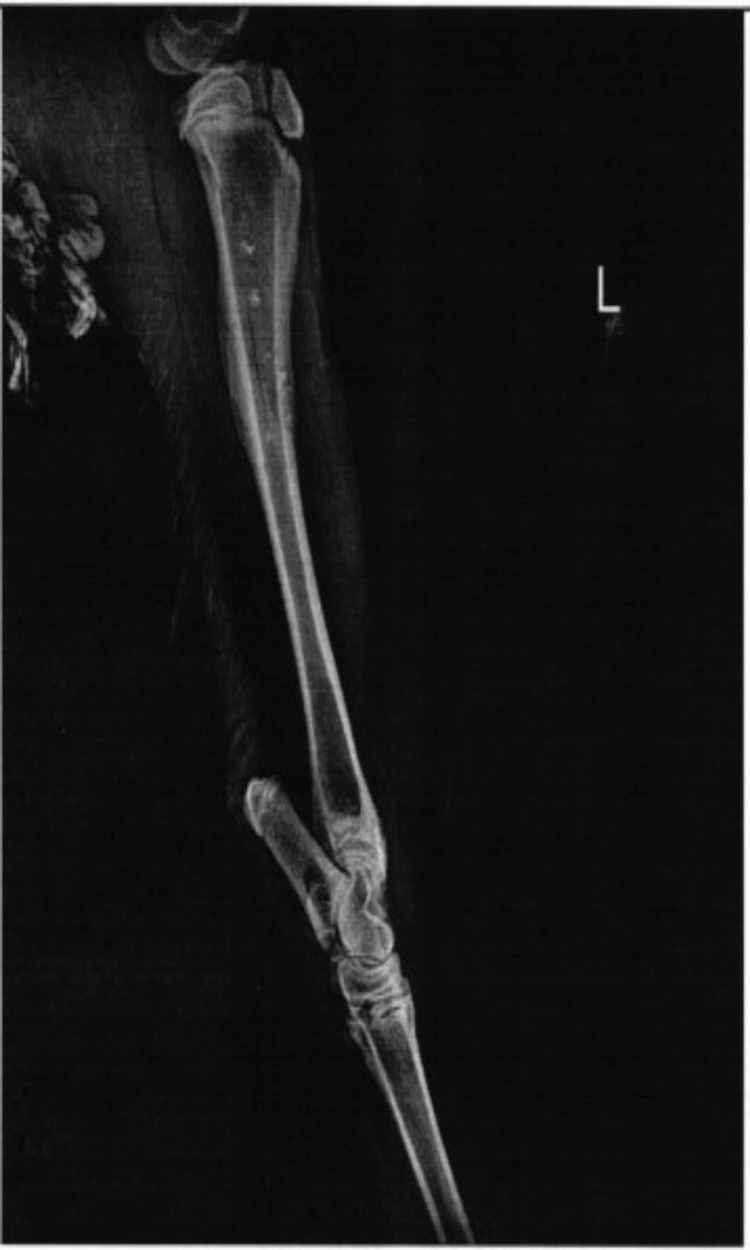
Mediolateral radiographic view 30 days after implantation with mild-to-moderate bone density.

**Figure 2:**
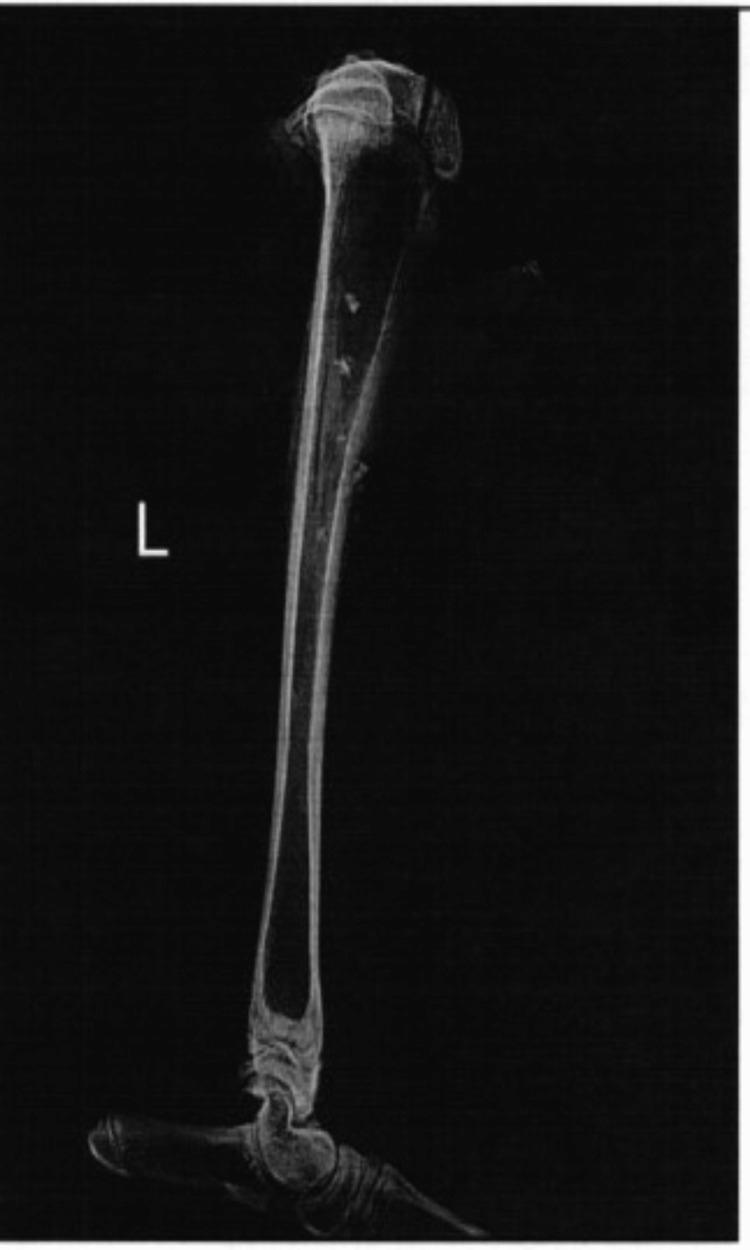
Mediolateral radiographic view 120 days after implantation. The bone defect was filled and new bone cells were organized.

**Figure 3:**
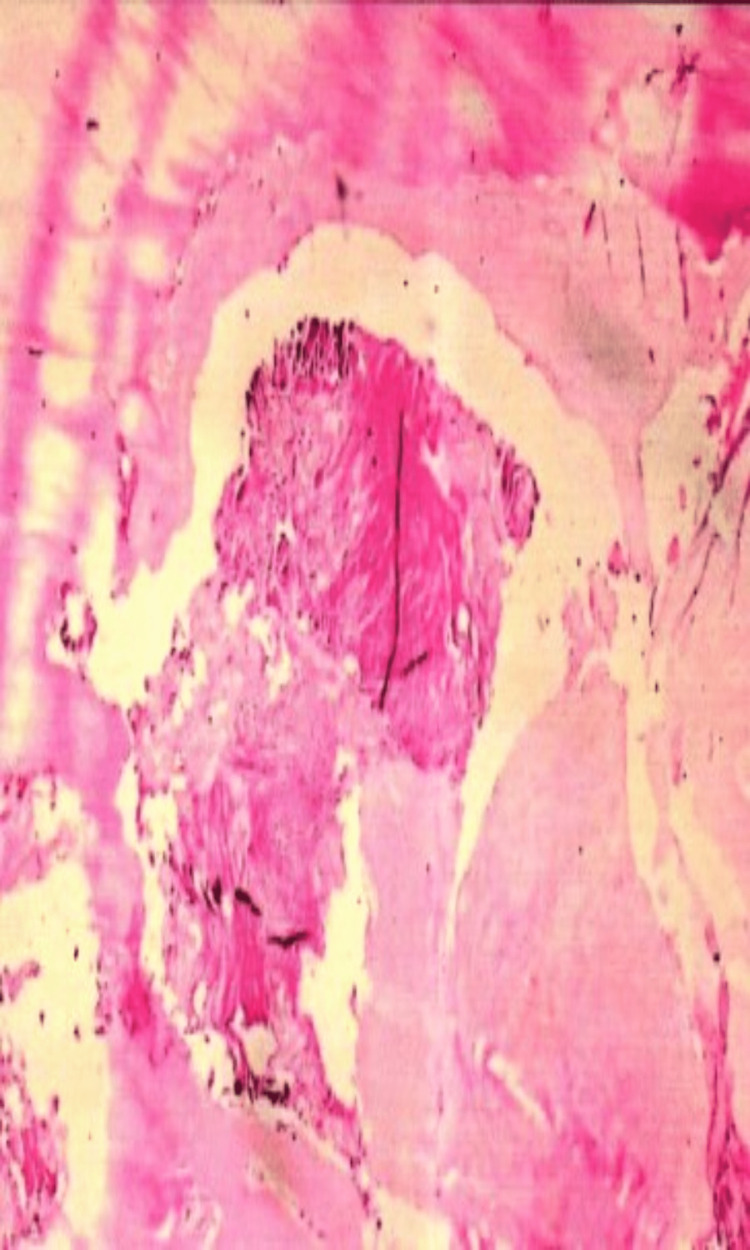
Bone implant graft material and the surrounding bone tissue.

**Figure 4:**
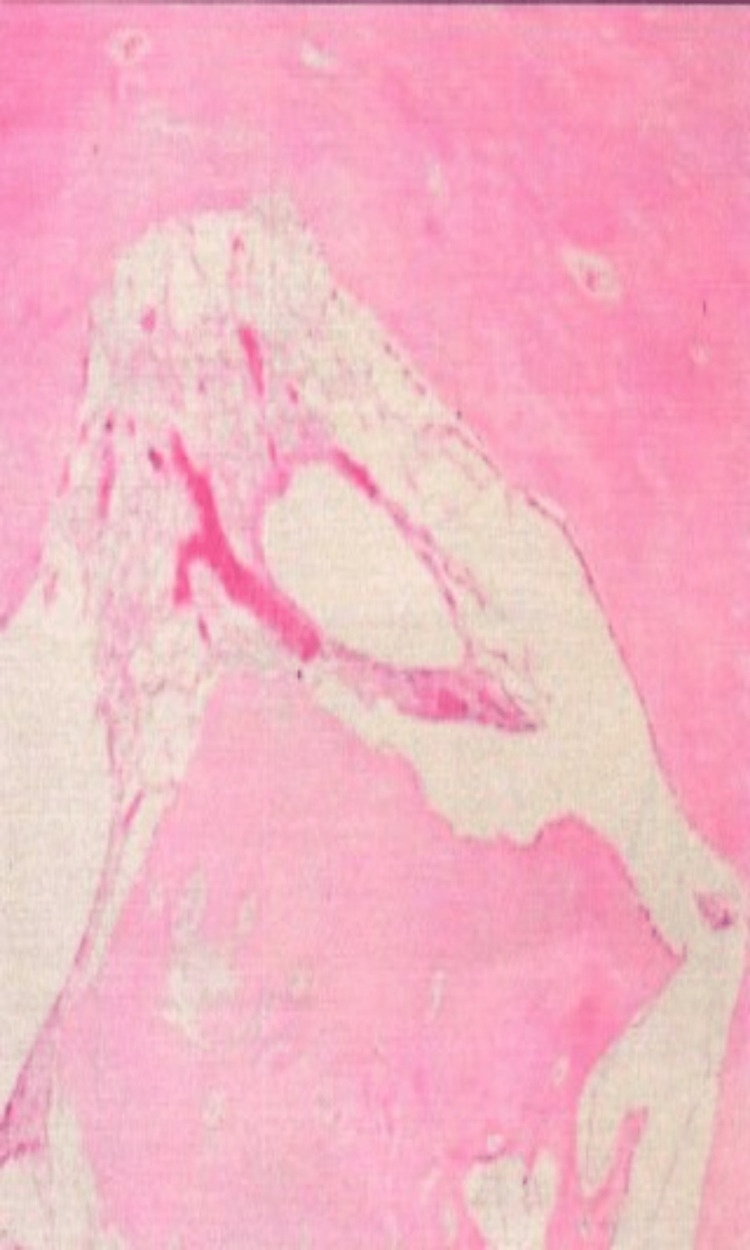
Fibrous tissue between bone and graft tissue.

**Figure 5:**
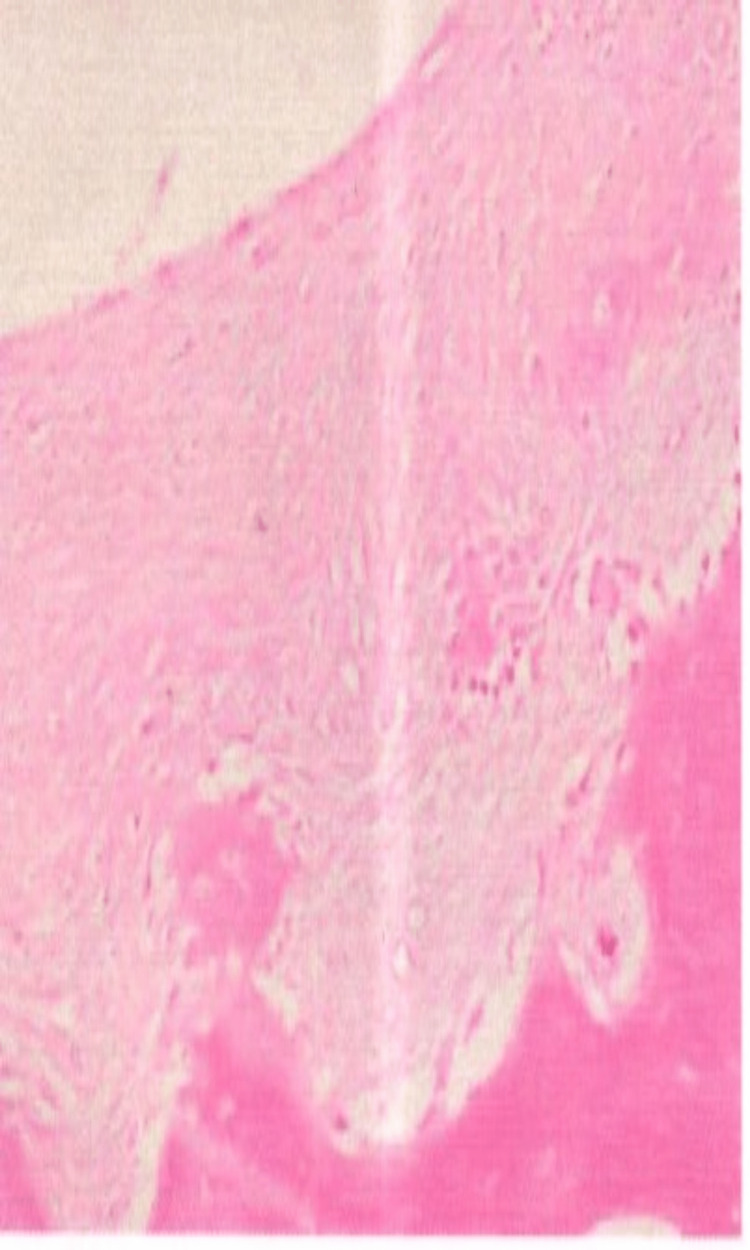
New remodeling bone tissue at the graft area.

**Figure 6:**
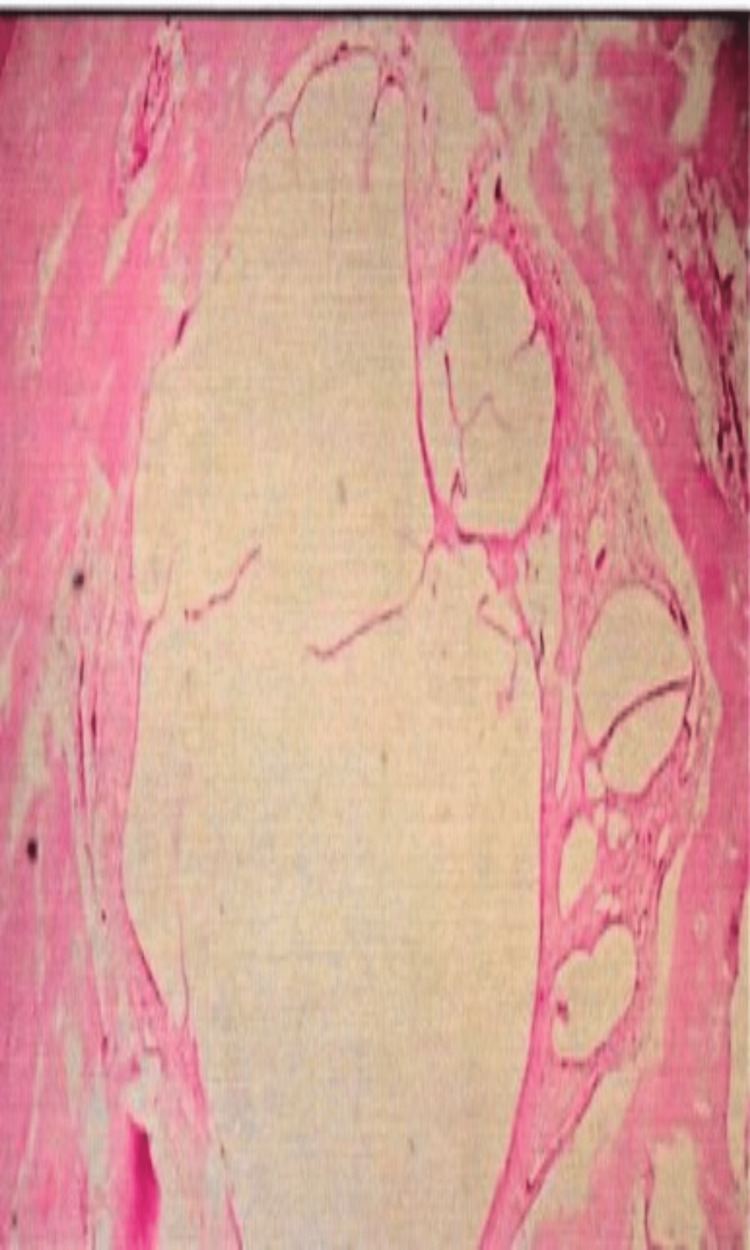
Control group: inadequate bone healing with minimal tissue reaction.

On radiographic view, the bone defect was filled with dense material, with minimal-to-moderate defect 30 days after implantation (Figure 1). At 120 days, the bone defect was closed to moderate mineralization without hypertrophic pathologic changes in the bone and soft tissues (Figure 2). Histological examination demonstrated a few macrophages and giant cells, with minimal fibrosis comparable to that in the control condition (Figures 3-6). The mean difference in the semi-quantitative scores between the graft and control condition was 2.33 (Table 1) (3.33-2.00 = 2.33). The level of irritation was based on the following categories: non-irritant (0.0-2.9), mild irritant (3.0-8.9), moderate (9.0-15.0), and severe irritant (>15.0).

**Table 1:** lSO 10993-6 (Annex 5): Semi-quantitative histopathological scoring in both groups.

Groups	Bioactive graft group			Control group		
Animal number	1	2	3	1	2	3
Inflammation	0	0	0	0	0	0
Polymorphonuclear leukocyte	0	0	0	0	0	0
Lymphocyte	0	0	0	0	0	0
Plasma cells	0	0	0	0	0	0
Macrophage	0	1	1	0	0	1
Giant cell	1	2	2	0	0	0
Necrosis	0	0	0	0	0	0
Neovascularization	0	0	0	0	0	0
Fibrosis	1	1	1	1	1	0
Fatty degeneration/infiltration	0	0	0	0	0	0
Total	2	4	4	1	1	1

## Discussion

This pilot study of a sheep implantation model demonstrated that the newly produced 45S5 bioactive graft is a safe and biocompatible material. The use of animal models, such as sheep, provides valuable insights into the efficacy and safety of bioactive glass-based materials for bone regeneration and clinical applications [15]. Bioactive glass has gained significant attention in the field of bone regeneration due to its unique properties and potential applications in orthopedics that have been found to induce osseointegration and bone healing in clinical and animal studies [16-21].

Wheeler et al. [16] demonstrated improved fracture healing, increased bone formation, and improved biomechanical properties with the use of bioactive glass in a rabbit osteotomy model. The bioactive glass group demonstrated increased active mineralization and increased biomechanical strength compared to the control group at four weeks, while both groups showed similar results at eight weeks after osteotomy. In our study, active mineralization was evident 120 days after implantation. However, biomechanical data were not recorded.

Regarding biocompatibility, Bellucci et al. [17] conducted an in vivo study utilizing a rat model to assess the biocompatibility of 45S5 bioglass scaffolds. The results demonstrated a lack of adverse reactions, including inflammation or cytotoxicity, suggesting good biocompatibility of the material. The ability of 45S5 bioglass to support cell adhesion, proliferation, and differentiation further supports its biocompatible nature. They reported the formation of new bone without any intervening fibrous tissue, consistent with the results of this study.

Granito et al. [18] compared the effects of biosilicate and bioglass 45S5 on tibial bone consolidation in rats. They assessed the biomechanical and histological aspects of bone healing 15 days after surgery. The results showed that both biosilicate and bioglass 45S5 enhanced bone consolidation compared to the control group. However, the biomechanical properties were significantly higher in the biosilicate group relative to bioglass, indicating better bone strength. On histological analysis, biosilicate and bioglass groups revealed increased bone formation and improved bone remodeling in both groups compared to the control group similar to our study.

Dehkordi et al. [19] investigated the radiological and histological effects of nano-bioglass and commercial bioglass on bone healing, bone regeneration, osseointegration, and histological changes in a rabbit fracture model. The results demonstrated that both nano-bioglass and commercial bioglass enhanced bone healing and promoted osseointegration. However, the nano-bioglass group showed better results in terms of radiological assessment, with greater bone density and bone healing than the commercial bioglass group. Histological analysis confirmed increased bone formation and osseointegration in both groups, supporting the positive effect of bioglass observed in our study.

Pugely et al. [20] investigated the influence of 45S5 bioactive glass incorporated into a standard hydroxyapatite-β-tricalcium phosphate-collagen (HA-TCP-collagen) as a bone graft substitute in the posterolateral fusion surgery of the rabbit spine. They assessed the fusion rates and evaluated the effects on bone healing. High fusion rates were observed at four and eight weeks with the use of the HA-TCP-collagen composite containing bioactive glass particles. The bioactive glass facilitated better bone formation and integration, leading to improved fusion outcomes in the rabbit spine. Histological evaluation demonstrated improved fusion rates and enhanced bone healing in the group treated with the HA-TCP-collagen composite containing bioactive glass compared to the group treated only with the HA-TCP-collagen composite. However, the finding of mild inflammation with macrophages and multinucleated giant cells is compatible with the findings of our study.

Moreira-Gonzalez et al. [21] created critical-size calvarial defects in rabbits and evaluated the effects of 45S5 bioactive glass on bone regeneration, integration, and histological changes. Contrary to our study, histological analysis demonstrated inflammatory reactions due to the cell-mediated biodegradation process. This result could be attributed to the fact that calvarial defects were created as critical defects of more than 15 mm in a rabbit model, which is very different from a sheep model.

Traditional bioactive glass production methods are divided into two different techniques, namely, sol-gel and melting. Micro-sized or nano-sized glass particles are generally obtained by the sol-gel method. On the other hand, with the melting method (melt-quenched), a partial amount of micro-sized glass particles are obtained, mostly in macro sizes, when no additional grinding process is used [22]. In addition, the melt-quenched method can separate the macro and microparticles obtained by the sieving process, and, ultimately, only macro-sized granular bioactive glasses can be obtained. Granito et al. [18] and Wheeler et al. [16] used melt-quenched techniques with different sizes (10 × 30 mm^2^ cylinder vs. 90-710 µm granules). Dehkordi et al. [19] used sol-gel and melt-quenched techniques for microsized granules without reporting the exact granule size. In our study, the bioactive graft was produced using the melt-quenched method without grinding to obtain macro-sized granules (1-3 mm). Although there are different production methods of bioactive granules, similar fusion rates and bone healing were observed in our study compared to previously reported studies [16,18,19].

The limitations of our study include the lack of histomorphometric analysis, micro-CT assessments, biomechanical analysis, and assessment of osseointegration and bone strength after filling of the bone defect using these bioactive granules. Further studies on 45S5 graft should include other options such as autografts and other types of allografts such as hydroxyapatite tricalcium phosphate as well as different formulations of bioglass such as BG-S53P4 to check their validity and their use as alternates to available bone grafts.

## Conclusions

Our study demonstrated the biocompatibility of 45S5 bioactive graft in a sheep model that justifies further preclinical use and highlights its ability to enhance bone consolidation. This material can be used as an alternative to available bone grafts.
